# Perception, Willingness, Barriers, and Hesitancy Towards COVID-19 Vaccine in Pakistan: Comparison Between Healthcare Workers and General Population

**DOI:** 10.7759/cureus.19106

**Published:** 2021-10-28

**Authors:** Muhammad Kashif, Iayla Fatima, Abdul Moiz Ahmed, Shajeea Arshad Ali, Roha Saeed Memon, Muhammad Afzal, Usama Saeed, Sana Gul, Junaid Ahmad, Farheen Malik, Mehreen Malik, Jawad Ahmed

**Affiliations:** 1 Anaesthesiology, Aga Khan University, Karachi, PAK; 2 General Surgery, St. Luke’s General Hospital, Killenny, IRL; 3 Internal Medicine, Dow University of Health Sciences, Karachi, PAK; 4 Dermatology, Jinnah Medical and Dental College, Karachi, PAK; 5 Internal Medicine, Liaquat University of Medical and Health Sciences, Jamshoro, PAK

**Keywords:** hesistancy, vaccine acceptance, prevention, pandemic, sars-cov-2, barriers, perceptions, pakistan, covid-19 vaccine

## Abstract

Background

Vaccine hesitancy has been a huge challenge in controlling preventable diseases. With the emergence of coronavirus disease 2019 (COVID-19) vaccines, it is vital to know their acceptance rates among the masses. No comparative data is available on the current subject from Pakistan yet. Therefore, this study aimed to evaluate the acceptance of a potential COVID-19 vaccine among the general population and healthcare workers (HCWs) of Pakistan, along with their perceptions and barriers to acceptance.

Methods

An online cross-sectional study was carried out in Pakistan from December 19, 2020, to January 10, 2021, using convenience sampling. A self-administered questionnaire consisting of 31 items was distributed after informed consent. Inclusion criteria consisted of HCWs and non-HCWs (general population) aged 18 years and above, residing in Pakistan. All analyses were done using Statistical Package for Social Sciences (SPSS) version 23.0 (IBM Corp., Armonk, NY, USA). Chi-square and T-test were used and a p-value of less than 0.05 was considered significant for all cases.

Results

Of the 404 respondents (n=196 general population and n=208 HCWs), 73.5% were willing to get a proven, safe, and effective COVID-19 vaccine if it was free of cost. This was reduced to only 64.3% if the vaccine was not free and had to be paid for. A total of 168 (41.6%) participants agreed to get vaccinated immediately, while 149 (36.9%) participants concurred to get it on a delayed basis. Eighty-seven (21.5%) participants refused to receive the COVID-19 vaccine, amongst which a significant majority (p<0.001) of the participants were from the general population. Doctors or scientists/scholarly journals were found to be the most trusted source of information (67.6%; n=273), while fear of unknown side effects (45.5%; n=184) was found to be the most common barrier towards COVID-19 vaccination. More than half (53.5%) participants believed that the vaccine is safe, effective, and has minimal side effects, amongst which a significantly large fraction (p<0.001) belonged to the HCWs.

Conclusion

The acceptance rate of a safe, effective, proven, and free COVID-19 vaccine was 73.5%. The fear of unknown side effects was the most common barrier to COVID-19 vaccine uptake. The general population demonstrated less knowledge, more false perceptions, and barriers to COVID-19 vaccine. Adequate measures should be taken to educate the masses about the COVID-19 vaccine, and its safety, and further studies are required.

## Introduction

The coronavirus disease 2019 (COVID-19), caused by severe acute respiratory syndrome coronavirus 2 (SARS-CoV-2), commenced a pandemic that still poses a significant threat to people, with symptoms ranging from pneumonia to multi-organ dysfunction. It has led to the downfall of economies and enumerated the burdens of mortalities and morbidities around the globe. As of October 27, 2021, a total of over 245 million COVID-19 cases have emerged, with more than 4.9 million deaths worldwide. Meanwhile, Pakistan has witnessed 1.27 million cases, out of which 28,392 people have lost their lives [1]. Although public health measures such as isolation, social distancing, and quarantine are effective in curbing the spread of COVID-19, various treatments are under usage for symptomatic patients, such as remdesevir, glucocorticoids, convalescent plasma transfusion, supplemental oxygen, and anticoagulants [2]. The only long-term solution, however, is immunization against SARS-CoV-2. Several vaccines, such as the mRNA-based vaccine developed by Moderna Inc., the non-replicating vector-based vaccine by CanSino Biologics, the DNA-based vaccine such as COVIGEN, nCov vaccine, Covigenix VAX-001, and protein subunit Novavax, to name a few, have been developed [3,4].

A global survey of 13,426 people was conducted by Lazarus et al. to find the acceptance rates of a potential COVID-19 vaccine across 19 countries. The results showed that participants from China among developed countries had the highest vaccine acceptance rate, i.e., 90% [5], while developing countries like Jordan showed an acceptance rate of only 28.4% [6]. This significant inequity in acceptance rates makes it harder for the global community to combat the pandemic.

No statistics about the comparative (between general population and HCWs) awareness and acceptance of the COVID-19 vaccine are available for Pakistan yet. Previous studies regarding awareness and acceptance of vaccines against different diseases in Pakistan have shown unfavorable results [7]. Bukhsh et al. showed that 24.4% of Pakistani parents were aware of the influenza vaccine; however, only 6.6% of them reported vaccinating their child against influenza [7]. Moreover, there has been an increase in the number of polio cases in Pakistan. In 2014, 328 polio cases were reported, compared to 58 in 2012 [8,9]. This correlates with Khan et al. study, which showed 84.8% of participants having a negative attitude towards polio immunization in two highly affected regions in Pakistan [10]. This indicates that despite the availability of vaccines, only a few get vaccinated, which is due to the possibility of it being against their religious beliefs, i.e., Halal-certified, and the myth that it causes the disease itself [8]. Owing to this, the eradication or at least the prevention of these diseases poses a big challenge.

Vaccination programs can only be successful if the acceptance rate is high [8,11]. It is important to note that at least 60-70% of the population needs to be vaccinated before the development of herd immunity [11]. Hence it is necessary to understand the individual preferences about vaccination. HCWs are often the high-risk population in the event of pandemics such as COVID-19. It is imperative that the HCWs are healthy enough to take proper care of the patients. The vaccine hesitancy in HCWs can not only put more strain on already strained health care systems but also make the general public to be more averse to COVID-19 vaccinations. This study aimed to evaluate the acceptance of a potential COVID-19 vaccine among the HCWs and the general population of Pakistan, along with their perceptions and barriers to acceptance.

## Materials and methods

Study design and duration

An online cross-sectional study was carried out amongst the population of Pakistan using convenience sampling. The study duration was from December 19, 2020, to January 10, 2021.

Sample size

OpenEpi.com was used to calculate the sample size for the study. Using the study by Lazarus et al. [5], which showed that global acceptance for the COVID-19 vaccine was 46.8%, a sample size of 383 was calculated for our population with a confidence interval of 95%.

Study population and inclusion criteria

A self-administered questionnaire was made using Google Forms. It was distributed to HCWs and the general population (i.e., non-healthcare workers) of Pakistan via social media platforms and direct e-mail solicitation. Questionnaires were distributed among national groups created on social media to spread COVID-19 information and concerns. The inclusion criteria consisted of two points, (1) current resident of Pakistan and (2) aged 18 years or older. The younger age group (<18 years) was not included.

Study tool

We conducted a detailed literature review to draft a questionnaire. The draft questionnaire was pilot tested among 30 participants (the results of which were not included in the study) and was sent to multiple senior researchers for evaluation, and all appropriate suggestions were incorporated in the questionnaire. Informed consent was obtained, confidentiality was maintained, and no personally identifiable information was collected or stored. The structured questionnaire (available in the Appendix) was finalized and consisted of 31 questions.

The questionnaire covered the following major aspects: (1) A brief explanation of the study, informed consent (2) Demographic variables such as age, gender, education level, marital status, parental status, family income, and comorbidities, (3) History of infection and awareness regarding SARS-CoV-2, (4) Vaccination status of the participants, (5) Awareness and willingness to take part in the ongoing COVID-19 vaccine trials, (6) Factors affecting the willingness of the study participants to get COVID-19 vaccine, (7) Inclination towards COVID-19 vaccine, (8) Sources of information regarding COVID-19 vaccination, (9) Factors influencing the decision to get COVID-19 vaccine, (10) Barriers towards COVID-19 vaccination, and (11) Perceptions and beliefs regarding COVID-19 vaccination.

For parts 1 through 9, only one and most appropriate response had to be marked, whereas for 10 (barriers towards COVID-19 vaccination) and 11 (perceptions and beliefs regarding COVID-19 vaccination), multiple choices could be marked. The individual questions can be read from the questionnaire in the Appendix section. The research data were stored on excel files and entered into statistical software, and were only accessible to researchers that were part of this study. No questions regarding the name of the participant or place of occupation were asked, and no identifiable information was collected through the questionnaire.

Statistical analysis

Statistical Package for the Social Sciences software (SPSS version 23.0; IBM Corporation, Armonk, NY, USA) was used to analyze all data. Results were drawn by descriptive statistics, and means with their standard deviation were presented for continuous variables such as age. Categorical variables were reported as frequencies and percentages. All responses were stratified as HCWs and the general population (non-healthcare workers). Chi-square test was used to establish associations between HCWs and general population groups and different study variables, e.g., demographics and willingness to get vaccinated. Independent sample t-test was used to find statistical significance between numerical (e.g., age) variables with categorical variables (e.g., willingness to get vaccinated). A p<0.05 was considered significant for all analyses. Incomplete questionnaires were excluded from the sample and were not analyzed. The columns of all tables denote study participants (total participants, HCWs, and general population) whereas rows denote other variables in the study such as different demographic variables and responses to different questions.

## Results

The current study included a total of 404 study participants, amongst which 196 were from the general population and 208 were HCWs. The mean age of the study population was 30.73 ± 12.58 years. Approximately 32% (n=128) of the study participants had pre-existing comorbidities (43%; n=55 from HCWs and 57%; n=73 from the general population), with diabetes being the most prevalent comorbidity followed by hypertension. The study population comprised predominantly of females (51.7%; n=209). Majority of study participants were graduates/post graduates (74.0%; n=299). Over half of participants were married (61.1%; n=247) and over one-third of participants were parents (35.9%; n=149). A significant difference was noted between the general population and HCWs in terms of gender, mean age, education, marital and parental status, monthly income, and some comorbidities. The demographics and clinical characteristics of the participants and individual p-values are outlined in Table 1.

**Table 1:** Socio-demographics and characteristics of the study sample. Number of healthcare workers: 208; number of participants from general population: 196 SD: standard deviation; PKR: Pakistani Rupee; *p-value of <0.05 was considered statistically significant. Statistical difference was measured between health care workers and general population group and calculated using chi-square and independent samples t test.

Variables	Total, N (%)	Healthcare workers, N (%)	General population, N (%)	P-value
Mean age (years) ± SD	30.73 ± 12.58	27.78 ± 11.19	33.86 ± 13.22	<0.001*
Gender				0.038*
Male	195 (48.3)	90 (46.2)	105 (53.8)	
Female	209 (51.7)	118 (56.5)	91 (43.5)	
Education				<0.001*
No formal education	49 (12.1)	0 (0.0)	49 (100)	
Primary	22 (5.4)	0 (0.0)	22 (100)	
Matric/ Intermediate	34 (8.4)	0 (0.0)	34 (100)	
Graduate/ Postgraduate	299 (74.0)	208 (69.6)	91 (30.4)	
Marital status				<0.001*
Married	157 (38.9)	59 (37.6)	98 (62.4)	
Unmarried	247 (61.1)	149 (60.3)	98 (39.7)	
Are you a parent?				<0.001*
Yes	145 (35.9)	53 (36.6)	92 (63.4)	
No	259 (64.1)	155 (59.8)	104 (40.2)	
Monthly family income (PKR)				0.049*
<50,000	151 (37.4)	67 (44.4)	84 (55.6)	
50,000-1,00,000	126 (31.2)	66 (52.4)	60 (47.6)	
>1,00,000	127 (31.4)	75 (59.1)	52 (40.9)	
Comorbidities				
Diabetes	54 (13.4)	20 (37.0)	34 (63.0)	0.022*
Hypertension	48 (11.9)	18 (37.5)	30 (62.5)	0.039*
Malignancy (benign or malignant)	6 (1.5)	3 (50.0)	3 (50.0)	0.942
Heart disease	28 (6.9)	14 (50.0)	14 (50.0)	0.871
Asthma/chronic lung disease	28 (6.9)	10 (35.7)	18 (64.3)	0.083
Kidney disease	22 (5.4)	13 (59.1)	9 (40.9)	0.463
Neurological disease	7 (1.7)	5 (71.4)	2 (28.6)	0.287
Autoimmune disease	13 (3.2)	7 (53.8)	6 (46.2)	0.863

History of infection and awareness regarding SARS-CoV-2

A large fraction of our study population was found to be well-aware of the existence of SARS-CoV-2 (93.6%; n=378) and deemed it a major problem for the community (85.4%; n=345). Compared to the general population, a significantly higher (p<0.001) number of HCWs were found to be cognizant of the presence of SARS-CoV-2 (97.6%; n=203 HCWs vs. 89.3%; n=175 from general population) and its effects on the community (n=196 HCWs vs. n=149 from general population).

**Figure 1:**
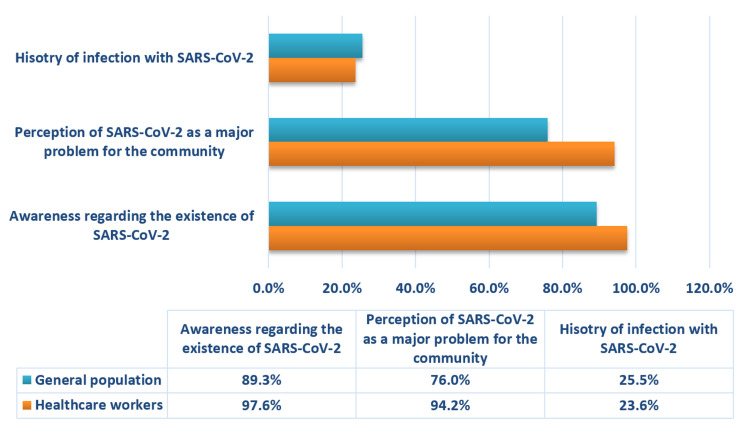
History of infection and awareness regarding SARS-CoV-2 among general population and healthcare workers SARS-CoV-2: Severe acute respiratory syndrome coronavirus-2

Around one-fourth of our study population (24.5%; n=99) had a history of infection of SARS-CoV-2. No significant association was found between the two groups (p>0.05). The findings are summarized in Figure 1.

Vaccination history of the participants

The study participants were surveyed regarding their vaccination status for diseases endemic in Pakistan, such as seasonal flu, tuberculosis, polio, and tetanus. More than half of our study population (65.3%; n=264) was found to be vaccinated, amongst which a significant majority (p<0.001) belonged to the HCWs (61.4%; n=162). The vaccination status of the participants' children was further evaluated. A vast majority of the participants (82.1%; n=119) were found to have vaccinated their children against endemic diseases. Amongst both groups, a significantly larger (p<0.001) number of HCWs (98.1%; n=52) had vaccinated their children as compared to the general population (72.8%; n=67).

Awareness and willingness to take part in the ongoing COVID-19 vaccine trials

A total of 55.9% of participants were found to be aware of the ongoing COVID-19 vaccine trials in Pakistan, amongst which a significant majority (p<0.001) were HCWs. A significantly smaller number of participants from the general population expressed an interest to be included in the ongoing vaccine trials (p=0.001). No significant association was established between people having a history of infection of COVID-19 or those having pre-existing comorbidities with the willingness to participate in the vaccine trials (p>0.05). The majority of the participants (71.3%) assented to prioritizing the at-risk populace and HCWs in the ongoing vaccine trials, as shown in Table 2.

**Table 2:** Willingness of the study participants to get COVID-19 vaccine. Total number of study participants: 404; Number of healthcare workers: 208; number of participants from general population: 196 COVID-19, Coronavirus disease 2019 Statistical difference was measured between health care workers and general population group.Cal culated using chi-square test; *p-value of <0.05 considered statistically significant

Questions	Total, N (%)	Healthcare workers, N (%)	General population, N (%)	P-value
Awareness regarding the COVID-19 vaccine trials going on in Pakistan	226 (55.9)	137 (60.6)	89 (39.4)	<0.001*
Willingness to take part in ongoing COVID-19 vaccine trials	150 (37.1)	93 (62.0)	57 (38.0)	0.001*
Assenting to the prioritization of at-risk populace and healthcare workers in vaccine trials	288 (71.3)	168 (58.3)	120 (41.7)	<0.001*
Willingness to get a proven, safe and effective COVID-19 vaccine free of cost	297 (73.5)	179 (60.3)	118 (39.7)	<0.001*
Willingness to get a proven, safe and effective COVID-19 vaccine on one's own expense	260 (64.3)	163 (62.7)	97 (37.3)	<0.001*
Willingness to get COVID-19 vaccine if recommended by a highly trustworthy source	312 (77.2)	182 (58.3)	130 (41.7)	<0.001*
Willingness to get COVID-19 vaccine if it’s mandatory in profession	321 (79.4)	185 (57.6)	136 (42.4)	<0.001*
Willingness to get COVID-19 vaccine if it’s required during domestic or international travel	314 (77.7)	182 (58.0)	132 (42.0)	<0.001*

Factors affecting the willingness of the study participants to get COVID-19 vaccine

We observed that a substantial majority of the participants (73.5%; n=297) were inclined towards getting a proven, safe, and effective COVID-19 vaccine if it was free of cost, while only 64.3% (n=260) of the participants agreed to pay for it. A vast majority (79.4%; n=321) of the participants were inclined to receive the vaccine if it was mandatory in their profession. Approximately three-fourths of the study participants consented to get vaccinated if required during domestic or international travel (77.7%; n=314) or recommended by a highly trustworthy source (77.2%; n=312). The stratification between the two groups is presented in Table 2. The preference for a domestic or imported vaccine was evaluated amongst both groups, which revealed that more than half of the study participants favored an imported vaccine (62.4%; n=252) over a domestic one (37.6%; n=152). A significantly (p<0.001) large number of HCWs preferred imported vaccine (n=142 HCWs vs. n=110 from general population) while participants from general population prioritized domestic vaccine (n=66 HCWs vs. n=86 from general population).

Inclination towards COVID-19 vaccine

**Figure 2:**
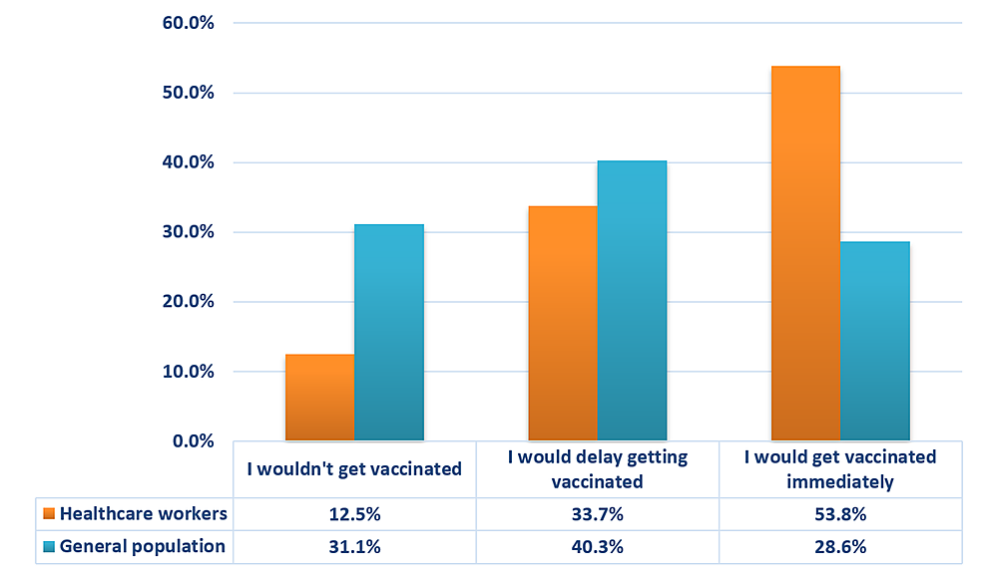
Participants' response towards an available vaccine for COVID-19. COVID-19: Coronavirus disease 2019

The participants were also surveyed upon whether they would like to get vaccinated with or without any further delay when the proven, safe and effective COVID-19 vaccine became available (Figure 2). A total of 168 (41.6%) participants (n=112 HCWs vs. n=56 from general population) agreed to get vaccinated immediately, while 149 (36.9%) participants concurred to get it on a delayed basis (n=70 HCWs vs. n=79 from general population). Less than a quarter of the study participants (21.5%; n=87) refused to receive the COVID-19 vaccine, amongst which a significant majority (p<0.001) of the participants were from general population (n=26 HCWs vs. n=61 from general population).

Association of demographics with inclination towards COVID-19 vaccine

We observed that the participants believing in the existence of SARS-CoV-2 (44.2%, n=167) and those who deemed it as a major problem for the community (47.5%, n=164) were significantly more inclined to get vaccinated immediately as compared to their counterparts (p<0.001). A significant difference (p<0.001) was found between the participants predisposed to get a proven, safe, and effective vaccine as compared to those who were not, younger people were more inclined towards vaccination. Around half of the female participants (55.4%, n=93) complied with getting immediately vaccinated as compared to their male counterparts (p>0.05). The difference among genders was not statistically significant. 

Participants with primary or no education were noted to be more inclined towards not getting vaccinated as compared to those with higher education (p<0.001). Moreover, we observed a significant preponderance of unmarried participants willing to get vaccinated without any delays as compared to their counterparts (122 vs. 46). Similarly, a significant majority of the parents included in our study vouched for getting either delayed (41.4%, n=60) or no vaccination (32.4%, n=47) as compared to their counterparts (p<0.001). The faction of our study population against the idea of getting vaccinated contained a majority of participants having a family income of below 50,000 Pakistani Rupee (PKR) (62.1%, n=54) while those wanting to get vaccinated immediately were a majority earning more than 100,000 PKR (40.5%, n=68) (p<0.001).

A total of 128 participants had comorbidities, amongst which 51 (39.8%) were willing to get vaccinated. And about 99 participants reported a history of COVID-19 infection, amongst whom 42 (42.4%) were willing to get vaccinated. No significant association was established between people having a history of infection of COVID-19 or those having pre-existing comorbidities with the inclination to get vaccinated on an immediate basis (p>0.05).

A significant association was established between the vaccination status of the participants and their children with the compliance towards COVID-19 vaccination. More than half of the study population vaccinated for seasonal flu, and endemic diseases were found to be more in favor of getting COVID-19 vaccination as soon as available as compared to those who were non-vaccinated (51.5%, n=136) (p<0.001).

Sources of information regarding COVID-19 vaccination

News was the most common source of information regarding the upcoming COVID-19 vaccination, followed by social media. It was observed that a significantly higher (p<0.001) number of participants from general population were unaware of vaccination.

As shown in Table 3, the participants were surveyed regarding their most trusted source of information regarding COVID-19 vaccination. Doctors or scientists/scholarly journals were found to be the most trusted source of information, followed by media. A significant majority (p<0.001) of participants from general population considered media to be a dependable source of information, while a predominantly higher number of HCWs relied on scholarly journals to get accurate details regarding vaccination.

**Table 3:** Sources of information and factors affecting the perception of COVID-19 vaccination. Number of healthcare workers: 208; number of participants from general population: 196 COVID-19, Coronavirus disease 2019 Calculated using chi-square test; *p-value of <0.05 considered statistically significant.

Variables/Questions	Total, N (%)	Healthcare workers, N (%)	General population, N (%)	P-value
From where you first heard about the upcoming COVID-19 vaccine?				<0.001*
Scholarly journals	20 (5.0)	14 (70.0)	6 (30.0)	
Social media	123 (30.4)	77 (62.6)	46 (37.4)	
News	138 (34.2)	67 (48.6)	71 (51.4)	
Magazines	3 (0.7)	3 (100)	0 (0.0)	
Doctors	36 (8.9)	19 (52.8)	17 (47.2)	
Friends or relatives	38 (9.4)	10 (26.3)	28 (73.7)	
I have not heard about it yet	46 (11.4)	13 (28.3)	33 (71.7)	
Who is your most trusted source of information regarding COVID-19 vaccine?				<0.001*
Parent/Guardian	27 (6.7)	13 (48.1)	14 (51.9)	
Religious preacher	9 (2.2)	1 (11.1)	8 (88.9)	
Political Figure	22 (5.4)	9 (40.9)	13 (59.1)	
Doctor or Scientists/Scholarly journals	273 (67.6)	160 (58.6)	113 (41.4)	
Media	73 (18.1)	25 (34.2)	48 (65.8)	
Do you believe that COVID-19 vaccination will protect you against COVID-19?				<0.001*
Yes	117 (29.0)	81 (69.2)	36 (30.8)	
No	40 (9.9)	14 (35.0)	26 (65.0)	
Maybe	247 (61.1)	113 (45.7)	134 (54.3)	
Has social media negatively influenced your decision on getting COVID-19 vaccination?				0.703
Yes	175 (43.3)	92 (52.6)	83 (47.4)	
No	229 (56.7)	116 (50.7)	113 (49.3)	
Do you think the negative attitude of surrounding people (like friends & family) towards vaccines discourages you to get the upcoming COVID-19 vaccine?				0.431
Yes	159 (39.4)	78 (49.1)	81 (50.9)	
No	245 (60.6)	130 (53.1)	115 (46.9)	

Factors influencing the decision to get COVID-19 vaccine

As presented in Table 3, only 29% of our study participants believed in the vaccine's efficacy. More than half of our study population seemed unsure about the protective effects of COVID-19 vaccination (61.1%), out of which a significant majority (p<0.001) belonged to the general population (54.3%).

Around 43% of our study population responded that social media influenced their decision concerning vaccination. Likewise, about two-fifths of the participants responded that their close acquaintances' negative attitude faltered their decision to receive the COVID-19 vaccine.

Barriers towards COVID-19 vaccination

Fear of unknown side-effects (45.5%) was found to be the most common barrier towards COVID-19 vaccination; however, no significant association was observed between the two groups, i.e., HCWs and general population (p>0.05). Around 35.4% of our study population felt that the vaccine was introduced recently, and its efficacy has not been proved yet; a significant majority (p=0.005) of these participants were HCWs. Similarly, 25.2% of the participants were hesitant towards the prospect of vaccination due to the lack of widespread trials, amongst which there was a significant preponderance (p=0.016) of HCWs.

A small proportion of our study sample (6.2%) considered COVID-19 to be a flu and deemed the vaccine to be unnecessary; a significant majority (p=0.001) of these participants were from the general populace (80%). One-tenth of our study participants (10.5%) believed that death is an unavoidable occurrence and vaccine could not prevent it; a significantly large (p=0.005) faction of these participants belonged to the general population (69%).

**Figure 3:**
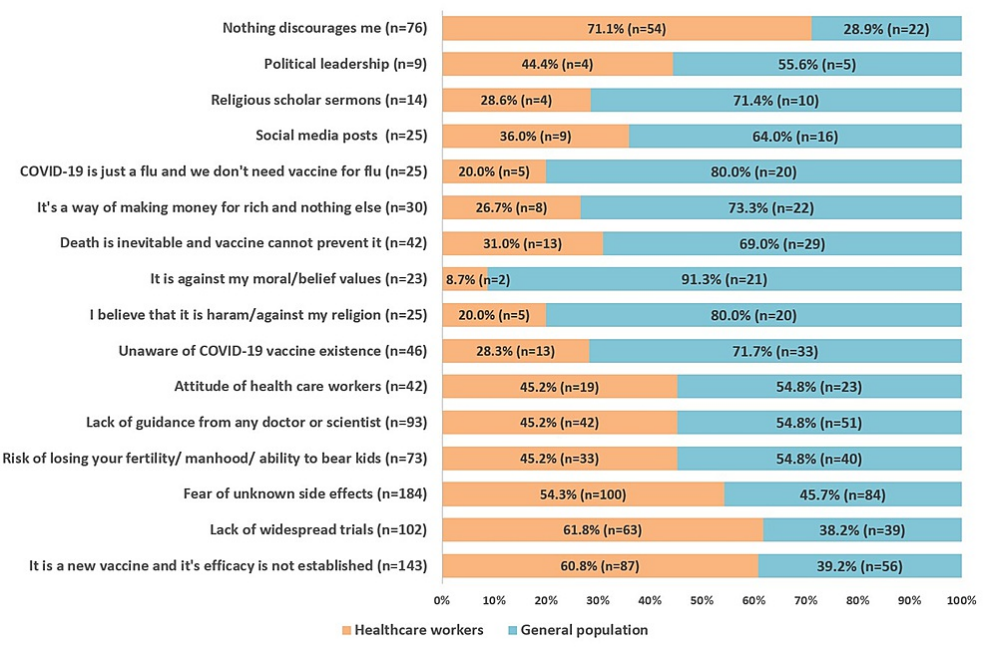
Barriers and discouraging factors relating to COVID-19 vaccination. COVID-19: Coronavirus disease 2019

Other discouraging factors towards COVID-19 vaccination were lack of guidance from a doctor or scientist (23%), risk of infertility (18.1%), the attitude of HCWs (10.4%), social media posts (6.2%), religious scholar sermons (3.5%), and political leadership (2.2%) as presented in Figure 3.

Perceptions and beliefs regarding COVID-19 vaccination

The study participants were surveyed regarding their beliefs and assumptions about the COVID-19 vaccine. A vast majority of the participants (53.5%) responded that the vaccine is safe, effective, and has minimal side effects, amongst which a significantly large fraction (p<0.001) belonged to the HCWs (68.1%).

**Figure 4:**
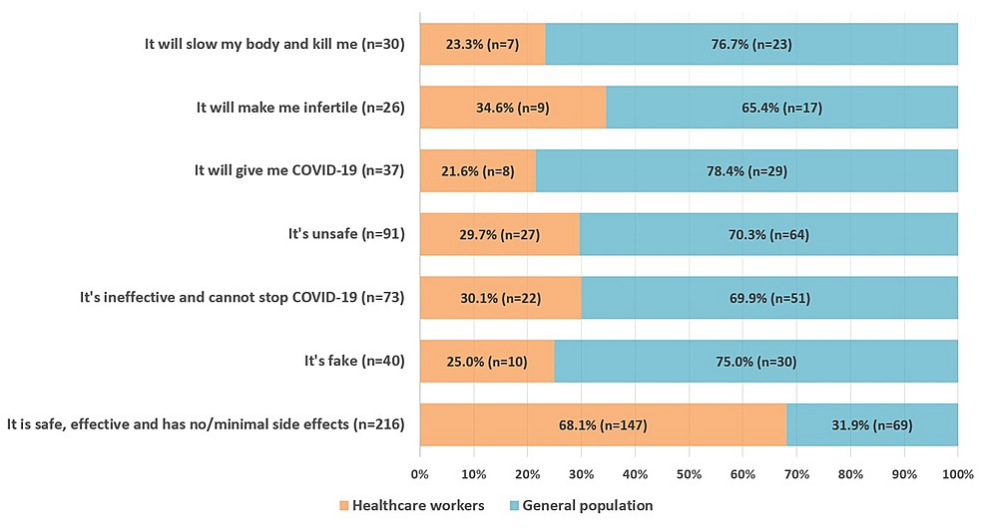
Perceptions and beliefs regarding COVID-19 vaccination. COVID-19: Coronavirus disease 2019

Around 9.9% of the study sample considered COVID-19 vaccination a complete sham; a significant majority (p<0.001) of these participants were from the general population (75%). Similarly, 18.1% (n=73) of the respondents felt that the vaccine was ineffective and cannot prevent disease transmission. Amongst both groups, a significantly lower (p<0.001) number of HCWs perceived the vaccine to be ineffective as compared to the general population. A significantly large (p=0.001) proportion of participants from the general population believed that the vaccine would gradually slow down their bodily functions and cause death (76.7%). The detailed results are illustrated in Figure 4.

## Discussion

This is one of the initial studies from Pakistan to assess the willingness of people to get vaccinated. Our study reported 73.5% of the participants were willing to get a safe, proven, and effective COVID-19 vaccine. Over half of the participants believed that the vaccine was safe and had minimal side effects, but fear of unknown side effects was a major barrier towards COVID-19 vaccination, as reported by 45.5% of the participants.

Among the two population groups in our study with a comparable history of COVID-19 infection, significantly more HCWs appreciated the existence and threat of SARS-CoV-2. This is consistent with a study by Simione et al., conducted in Italy with 353 participants, with similar population groups (HCWs and general population), HCWs reported greater knowledge, perceived higher risk, and expressed greater worry regarding COVID-19 infection compared to general population [12]. As HCWs deal with COVID-19 patients, their knowledge about the disease and serious manifestations of SARS-CoV-2 infection (such as coagulopathy, renal, cardiac and nervous system involvement), is higher compared to general population. In another cross-sectional survey of 630 adults in US by Wolf et al., individuals with low health literacy were reportedly less worried and firmly believed that they are immune to COVID-19 [13]. These results comply with the fact that HCWs being directly involved in public health naturally recognize the presence and menace of emerging infections earlier than others. In addition, HCWs are less likely to fall for the disease-related myths and misconceptions due to their inclination towards and active participation in the scientific medical literature. In a meta-analysis the predictors of intention and behavior, it was concluded that awareness of the disease is imperative in establishing preventive beliefs, promoting positive attitudes and behaviors, which subsequently pave the way for better efficacy of disease coping strategies [14]. Knowledge of disease and its adverse manifestations (COVID-19 in our case) make individuals more aware towards its prevention, and hence more drawn towards vaccination (prevention). Consistently, HCWs could be expected to show positive attitudes and behaviors owing to greater health literacy levels. These results hold true as when baseline vaccination status of our participants was assessed, HCWs were reportedly more prompt in getting themselves and their children vaccinated against the endemic diseases of Pakistan. Furthermore, vaccination as a requirement for different aspects of life, such as job or travel, can also be a motivating factor to get vaccination for some. Lack of intent for vaccination itself may be driven by lack of knowledge of COVID-19, this aspect has not been covered by our study and further research is warranted on it.

General population respondents constituted a major part of the population unaware of the COVID-19 trials, which was otherwise a small proportion. An Ipsos survey concluded a staggering 50% of Pakistanis were unaware of the ongoing COVID-19 vaccine trials [15]. In contrast, more than half of our study participants reported awareness; however, less than half showed interest in being inducted into these trials. In both the cases, general populace contributed a minor percentage to the overall number. Vaccine awareness and inclination towards participation in vaccine trials seemed to be an outcome of a better understanding of COVID-19, as it was more prevalent among HCWs. Secondly, the resources preferred for vaccine-related information could have influenced participants' knowledge and attitudes.

The most common sources used were news and social media whereas, doctors, scientists, and scholarly journals were labeled as the most trusted ones for the overall population and particularly HCWs. The source of information and trusted source differ among HCWs as they are more aware of the extent of false news circulating on social, and as most educated individuals are aware that authentic scientific facts are being disseminated by journals. In contrast, general population relied predominantly on social media as a trustworthy source of information. Social media is well-known for providing a means to circulate false rumors and construct misconceptions [16], and this practice has heightened during the pandemic [17] whereas, doctors, scientists, and scholarly journals challenge and dispel these misconceptions with scientific reasoning [18]. Thus, the general population in our study is also more likely to be misguided about the gravity of the situation and, likewise, adopt negative attitudes and behaviors to the introduction of vaccine and vaccine trials. An Arabic study also concludes higher vaccine conspiracy belief scores in participants relying exclusively on social media platforms for vaccine-related information [6]. While attempting to neutralize misleading narratives regarding COVID-19 origin, a former study had already suspected resistance against upcoming COVID-19 vaccination programs owing to these conspiracy theories [11]. Pakistan is particularly susceptible to fall for such theories since failure to eradicate polio is also attributed to these misconceptions [11].

Consistently, HCWs acknowledged the safety and efficacy of the COVID-19 vaccine, whereas negative perceptions were witnessed in the general population. About one-third (35.4%) considered the vaccine ineffective in the containment of COVID-19 whilst being unsafe itself and a direct source of SARS-CoV-2 infection. Perceptions analogous to the latter have been known to obstruct and fail anti-polio campaigns in the country [19]. Furthermore, a majority of the general population also believed that the vaccine would slow down body functions, including fertility, ultimately causing death. Dr. Mohsin Ali leading the Chinese COVID-19 vaccine trial in Pakistan, reported coming across similar concerns among trial volunteers [20]. Lastly, some recruits of our study outrightly labeled the vaccine as a fraud. The endeavor to tackle the spread of COVID-19 effectively remains futile in the absence of the right grasp of perceptions and beliefs presiding among the masses. The most important facet of vaccination is more about having a fully approved vaccination rather than the vaccine under trials or vaccine administered under emergency use of approval [11].

When willingness towards getting vaccinated was assessed, a majority affirmed that they would get vaccinated if it were proven safe and effective; however, it being free of charge seemed to attract an even larger group. These results add to a meta-analysis of global surveys in which a striking 68.4% of the world population showed willingness to get immunized against COVID-19 [21]. In the Ipsos survey, 60% of the respondents reported willingness, whereas, in a Gallup Pakistan poll, an estimated 37% of the population outrightly refused to get the vaccine [15]. Considering a positive history of vaccine hesitancy in Pakistan, 37% is an alarming proportion for the country and for the world that is depending on universal vaccine coverage to curb the spread of the virus, the pollster stated [20]. Slightly higher willingness to get vaccinated in our study compared to the Ipsos survey is maybe due to disproportionate number of HCWs that are more inclined towards getting vaccinated. Another majority of our participants declared that they would consider getting vaccinated if it were mandatory in their profession or during travel, while for others, a trustworthy recommendation was a deciding factor.

HCWs preferred an imported vaccine whereas the general population favored the domestic one. This divide seemed to have been influenced by the so-called 'West phobia' mindset inherent amongst Pakistan’s general population [20]. HCWs had the ease of access to the vaccine and were also reminded to get themselves vaccinated immediately, whereas the general population either opted for delayed immunization or none at all. This inclination seemed to be influenced by social media and the negative attitudes of people around our general population group. Thus, the availability of a safe and effective vaccine per se does not guarantee herd immunity; instead, vaccine hesitancy seems to be an imminent threat carrying the potential to nullify the outcome of immunization drive [22]. Vaccine hesitancy has been attributed to a 3 C's (confidence, complacency, and convenience) model comprising the lack of confidence in vaccine and its providers, complacency towards the need for vaccination, and inconvenience due to physical unavailability, geographical inaccessibility, and unaffordability [23]. In our study, HCWs preferred imported vaccines, whereas the general population was more inclined towards domestic ones. Hence, during the initial stages of vaccine drive, HCWs readily accepted vaccines imported in Pakistan. The percentage of willingness dropped if a certain payment had to be made for it, showing participants found free vaccination to be more convenient than a paid one.

Myths, misconceptions, and false perceptions fuel vaccine hesitancy and opposition that can hamper the desired outcomes of vaccination programs as exemplified by other vaccine-preventable infections like flu [24]. The key to demolishing vaccine hesitancy is addressing the barriers that wavered participant’s decision to get immunized. Fear of unknown side-effects was the most common overall barrier among all participants whether willing or unwilling to get vaccinated, whereas HCWs were more concerned over the lack of proven efficacy due to its recent introduction and subsequent trial paucity. Similar concerns over the vaccine’s efficacy and safety profile were expressed by the Ipsos survey participants [15]. Unawareness and misconceptions about the vaccine proved to be critical hesitancy factors among our study's general population. Some disregarded the necessity of the vaccine either because they considered COVID-19 to be a flu or denied the role of vaccine as lifesaving while the rest claimed it to be merely a lucrative business for the rich albeit, there was a small group that stated no discouraging factors. In addition, some labeled social media, religious scholar sermons, politics, and the myths about the loss of fertility as factors discouraging vaccination. Congruently, a former study reveals the involvement of political influences, cultural and religious beliefs in vaccine repulsion [25]. Similar to our study, Saied et al. reported barriers towards COVID-19 vaccination among students of a developing country, Egypt [26]. Participants reported barriers namely doubt of vaccine safety and effectiveness, fear of unknown side effects, fear of nano-chips in vaccine, lack of awareness, and financial cost [26]. Furthermore, lack of adequate guidance from a health professional or their inappropriate attitude reportedly hindered participants' inclination towards vaccination. However, these statements could be another outcome of conspiracy dialogue fostering mistrust in HCWs and their recommendations [27]. 

Recent studies which have been done to primarily determine the rate of vaccine acceptance and hesitancy in Pakistan have produced comparable results. One of these has approximated vaccine acceptance percentage around 53% [28]. But has not analyzed individual barriers towards COVID-19 vaccine hesitancy and has not evaluated the vaccination status of the population against other endemic infectious diseases in Pakistan. Our study aims to overcome these limitations and has found that the fear of unknown side effects (45.5%) is the most common barrier towards COVID-19 vaccination. About 65.3% were found to be vaccinated against other endemic infectious diseases in Pakistan such as polio or tetanus.

Another study approximates vaccine acceptance rate to be 70% in the population of Pakistan [29]. This study also reports a quite similar finding of HCWs/doctors being the most reliable source of information in view of the respondents. Our study addresses a limitation in this study which includes computation of awareness level of COVID-19 in the population that turned out to be 93.6%.

Limitations and recommendations

Some limitations should be considered. Considering the observational, cross-sectional nature of this study, the associations reported may not be causal. Self-administered questionnaires could have resulted in social desirability bias. However, bias was reduced by ensuring participants’ anonymity.

Employing convenience sampling technique, invitation to participate in the survey was made by e-mail and social media platforms; thus, people not reachable with these channels were not included in the study, which may have led to some selection bias. The general population was more prone to this bias as those individuals with no access or habit to the new technologies (older people and/or from remote areas) were not represented in those statistic results. Moreover, participation was voluntary, and thus people more sensitive to the COVID-19 vaccination topic may have a higher probability of responding and being included in the study. In addition, our results offer insights into the public’s perception of vaccines; a large-scale research needs to be done at a national level for more robust results. Finally, our study primarily evaluated awareness regarding vaccine trials and false perceptions regarding the COVID-19 vaccine. There is a need to assess the knowledge of vaccines' well-established scientific limitations among the participants as well [30]. This is important as a different approach is required to address these limitations that are inevitable and cannot be controlled for. Counseling could be one of the key steps employed in these situations to build the public's confidence over a higher benefit-risk ratio profile of the vaccine.

Our manuscript calls for further research from all over Pakistan as we report concerning findings. These studies would help determine overall awareness regarding vaccines which would then pave the way for focused efforts to fill in the knowledge gaps. The efforts can include introduction of information related to vaccine-preventable diseases in the curriculum or mass advertisement campaigns. Poor knowledge is alarming, especially for a country like Pakistan that suffers from an acute shortage of medical resources and has already exhausted most of these in managing the growing number of COVID-19 cases. Such a country could definitely benefit from a vaccine that efficiently tackles the infection spread. Improved understanding of the vaccine would itself give rise to favorable attitudes and practices thereafter; however, to manually improve the perceptions and behaviors, factors attributed to vaccine hesitancy could directly be evaluated in these studies and addressed accordingly.

## Conclusions

Overall, the general population demonstrated less knowledge, more false perceptions, and barriers to COVID-19 vaccine. This strengthens the need to take measures and to address the rumors and conspiracy theories igniting distrust in the efficacy and safety of the vaccine. Further studies in Pakistan could yield a holistic conclusion on the overall knowledge levels and perceptions towards the vaccine. These comprehensive findings could help formulate a plan at the national level by an invited collaboration of healthcare and educational institutes, public health departments, and social media platforms to spread authentic scientific knowledge regarding the vaccine and promote confidence in its reliability.

## Appendices

Online questionnaire for study

Introduction and consent

Greetings everyone!

This study is being conducted by researchers from <<Institute Name>>. The purpose of this research is to find the opinion and willingness about vaccination, and the barriers that prevent them from vaccination. Your participation in this survey is voluntary and will take approximately 5 minutes of your time. Any information that you mention will be kept confidential and you can withdraw at any time. There will be no credit or monetary compensation for participation. The results of this research will be published in a scientific journal. Please only fill this questionnaire if you are 18 years or above and are a resident of Pakistan. In case of any query, please contact the details provided. By clicking next, you agree to be a part of this research.

Thank you for your time.

If you have any query, please contact <<Author name>>, <<Email and phone number>>.

Demographic biodata

1. Gender

·       Male

·       Female

2. Age (in years)_____________

3. Education

·       No formal education

·       Primary

·       Matric(O levels)/Intermediate(A levels)

·       University student/Postgraduate

4. Marital Status

·       Married

·       Unmarried

5. Are you a parent?

·       Yes

·       No

6. What is your monthly family income? (PKR)

·       <50,000

·       50,000-100,000

·       >100,000

7. Are you a Health-care Worker?

·       Yes

·       No

8. Do you have any disease/medical condition?

·       Yes

·       No

9. If yes, then select all that applies:

·       Diabetes

·       Hypertension

·       Cancer

·       Heart disease

·       Asthma/chronic lung disease

·       Kidney disease

·       Neurological disease

·       Autoimmune disease

History of infection and awareness about SARS-CoV-2

10. Do you believe that Coronavirus exists?

·       Yes

·       No

11. Have you ever been infected with COVID-19?

·       Yes

·       No

12. Do you think it is a major problem for the community?

·       Yes

·       No

Vaccination status of participants

13. Have you ever been vaccinated for seasonal flu/TB/Polio/Tetanus(DPT) etc?

·       Yes

·       No

·       Maybe

14. Did you vaccinate your children for seasonal flu/TB/Polio/Tetanus(DPT) etc?

·       Yes

·       No

·       I am not a parent/guardian.

Awareness and willingness to take part in vaccine trials

15. Do you know that vaccine trials are going on in Pakistan and participants have shown no major side effects yet?

·       Yes

·       No

16. Are you willing to participate in COVID-19 vaccine trials going on?

·       Yes

·       No

17. Do you think it is fair that the first round of COVID-19 vaccine is being given to health care workers and people at risk ?

·       Yes

·       No

Factors affecting the willingness of the study participants to get COVID-19 vaccine

18. Will you be willing to get a proven, safe and effective COVID-19 vaccine once it's available for free?

·       Yes

·       No

19. Will you be willing to get the same vaccine if your most trustworthy source e.g your sibling/parent/family member/friend or any leader or any doctor recommends it to you?

·       Yes

·       No

20. Will you get the vaccine if your job makes it compulsory?

·       Yes

·       No

21. Will you get the vaccine if international or domestic travelling requires it?

·       Yes

·       No

22. Will you be willing to get the same vaccine if it is NOT free?

·       Yes

·       No

23. If you have to get vaccinated, would you rather go for a domestic vaccine or an imported one?

·       Domestic

·       Imported

Inclination towards COVID-19 vaccine

24. If a vaccine is available, will you get it on an immediate basis or delay your vaccination?

·       Immediately

·       Delayed

·       I won't get the vaccination

Source of information regarding COVID-19 vaccination

25. From where did you first hear about the upcoming COVID-19 vaccines?

·       Scholarly Journal e.g., NEJM, JAMA, Nature, etc.

·       Social Media (Facebook/Twitter/Instagram/Whatsapp)

·       News

·       Magazines

·       Doctors

·       Friends or relatives

·       I have not heard about it yet

26. Who is your MOST TRUSTED source of information regarding COVID-19 vaccine?

·       Parent/Guardian

·       Religious preacher

·       Political Figure

·       Doctor or Scientists/Scholarly Journals

·       Media

Factors influencing the decision to get COVID-19 vaccine

27. Do you believe that COVID-19 vaccines will protect you against COVID-19?

·       Yes

·       No

·       Maybe/Unsure

28. Has social media influenced your decision on getting COVID-19 vaccination?

·       Yes

·       No

29. Do you think the negative attitude of surrounding people (like friends & family) towards vaccines discourages you to get the upcoming COVID-19 vaccine?

·       Yes

·       No

Barriers towards COVID-19 vaccination

30. What discourages you from getting upcoming COVID-19 vaccine? Select all that applies:

·       It is a new vaccine and its efficacy is not established

·       Lack of widespread trials

·       Fear of unknown side effects

·       Risk of losing your fertility (or manhood or ability to bear kids)

·       Lack of guidance from any doctor or scientist

·       Attitude of health care workers

·       Unaware of COVID-19 vaccine existence

·       I believe that it is haram/against my religion

·       It is against my moral/belief values

·       Death is inevitable and vaccine cannot prevent it

·       It is a way of making money for rich and nothing else

·       COVID-19 is just a flu and we don't need vaccine for flu

·       Social media posts

·       Religious scholar sermons

·       Political leadership

·       Nothing discourages me

Perceptions and beliefs regarding COVID-19 vaccination

31. What is/are your belief(s) about the coronavirus vaccine? Select all that applies:

·       It is safe, effective and has no/minimal side effects

·       Its fake

·       Its ineffective and cannot stop COVID-19

·       Its unsafe

·       It will give me COVID-19

·       It will make me infertile

·       It will slow my body and kill me

Thank you for your participation in this research.

Note:

The questionnaire can be used for research purposes, provided that proper credits be given to the journal and authors by citing the study.
